# Correction: Cognitive & motor skill transfer across speeds: A video game study

**DOI:** 10.1371/journal.pone.0303465

**Published:** 2024-05-02

**Authors:** The PLOS ONE Staff

In the Data evaluation subsection of the Methods, there is an error in the partial equation in the paragraph under equation (6). The *n* is missing. Please view the correct equation here: $n\times log\frac{RSS}{n}$

The heading for the second column in Table 6 is incorrect. Please see the correct Table 6 here:

**Table:** 

	*LMEM*			*Multi-level correlations*		
	*β*	95% CI		*r*	95% CI	
Entropy	-6510301^***^	(-9229947,	-3862927)	-0.63^***^	(-0.75,	-0.48)
Log CV ISI	-1606117^**^	(-2703241,	-405376)	-0.62^***^	(-0.74,	-0.47)
Periodicity	-2743	(-8964,	4027)	-0.22^*^	(-0.42,	0.00)
Regularity	2791891	(-1184017,	7283639)	0.38^***^	(0.17,	0.55)
Adjusted *R*^*2*^	0.80					

^***^ *p* < .001

^**^ *p* < .01

^*^ *p* < .05; *β*s refer to linear estimates and *r* is a multilevel correlation coefficient [77].

The publisher apologizes for the errors.

The caption for Fig 1 was included in the article text instead of as a caption. Please see the complete, correct Fig 1 caption here.

**Fig 1:**
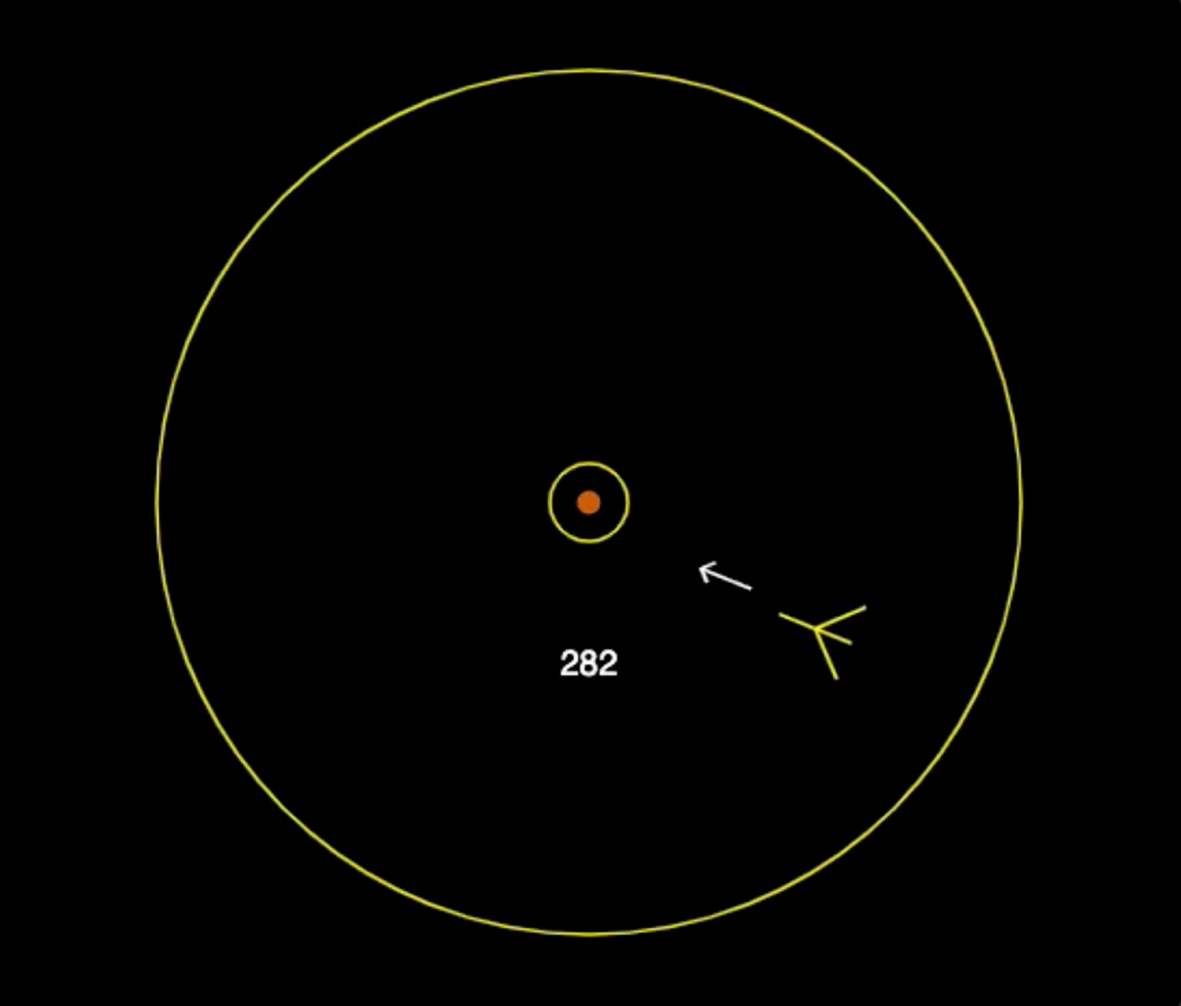
Illustration of the *Auto Orbit* video game interface. A spaceship is flying in a clockwise orbit around a balloon. The player needs to learn to adjust the spaceship’s aim and shoot periodically to inflate the balloon and burst it with a double shot. Reprinted from [67] under a CC BY license, with permission from Terrence C. Stewart, original copyright 2020.
