# Supplementary figures and images for: Cognitive & motor skill transfer across speeds: A video game study
Source: PLoS One. 2021 Oct 12;16(10):e0258242. doi: 10.1371/journal.pone.0258242 (PMC8509974; doi:10.1371/journal.pone.0258242)

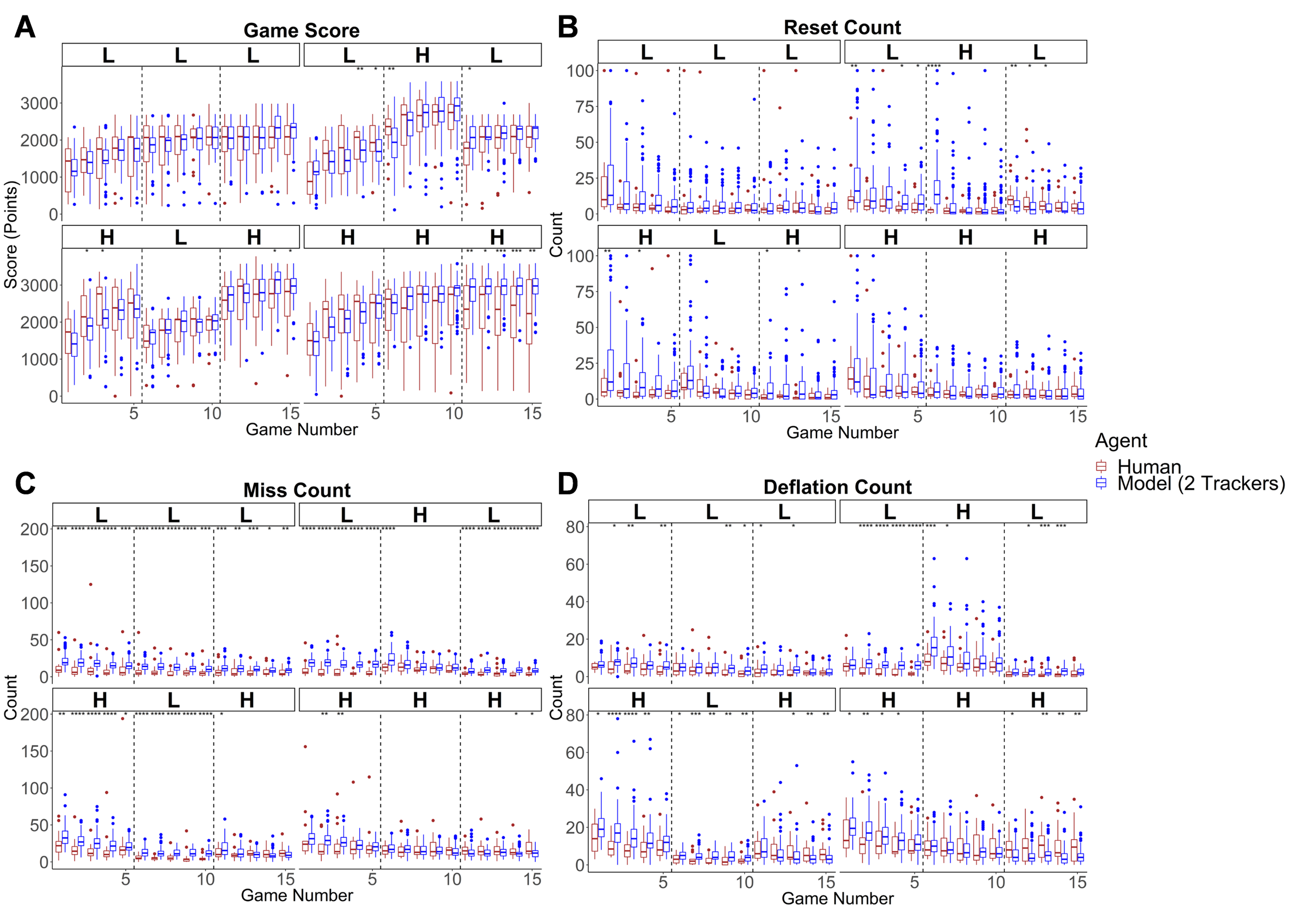

Supplement: S1 Fig — (A) Game score distribution over the 15 games across agents. (B) Reset count distribution over the 15 games across agents. (C) Miss count distribution over the 15 games across agents. (D) Deflation count distribution over the 15 games across agents. Boxplots indicate the median, 1st quartile and 3rd quartile. Across all plots, humans are shown in brown and ACT-R models are shown in blue. Performance measures solely include data from the ACT-R model with two trackers and one temperature. **** p < .0001; *** p < .001; ** p < .01; * p < .05. (TIF) [file pone.0258242.s001.tif]

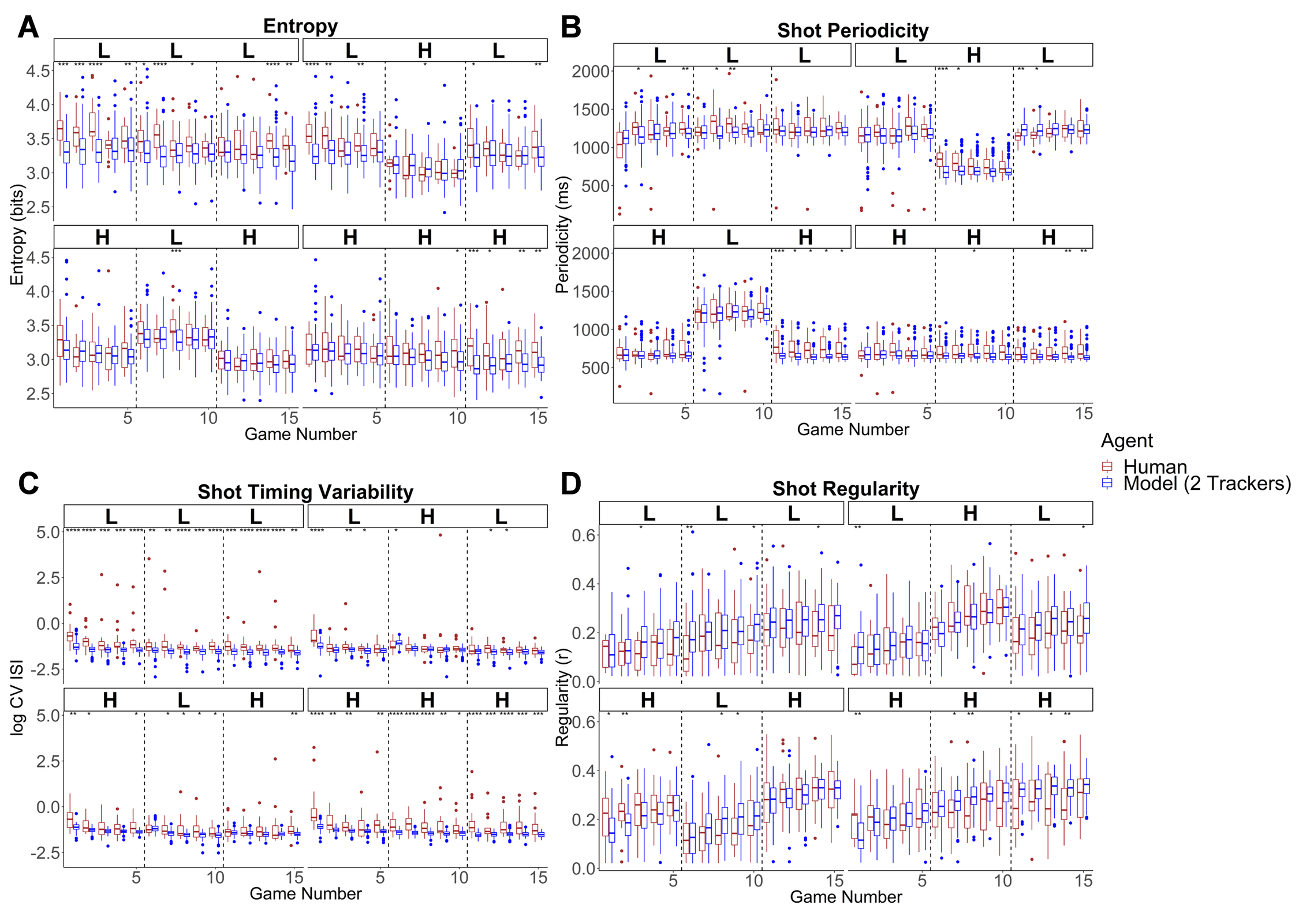

Supplement: S2 Fig — (A) Entropy distribution over the 15 games across agents. (B) Shot periodicity distribution over the 15 games across agents. (C) Shot timing variability distribution over the 15 games across agents. (D) Shot regularity distribution over the 15 games across agents. Boxplots indicate the median, 1st and 3rd quartiles. Across all plots, humans are shown in brown and ACT-R models are shown in blue. Motor learning measures solely include data from the ACT-R model with two trackers and one temperature. **** p < .0001; *** p < .001; ** p < .01; * p < .05. (TIF) [file pone.0258242.s002.tif]

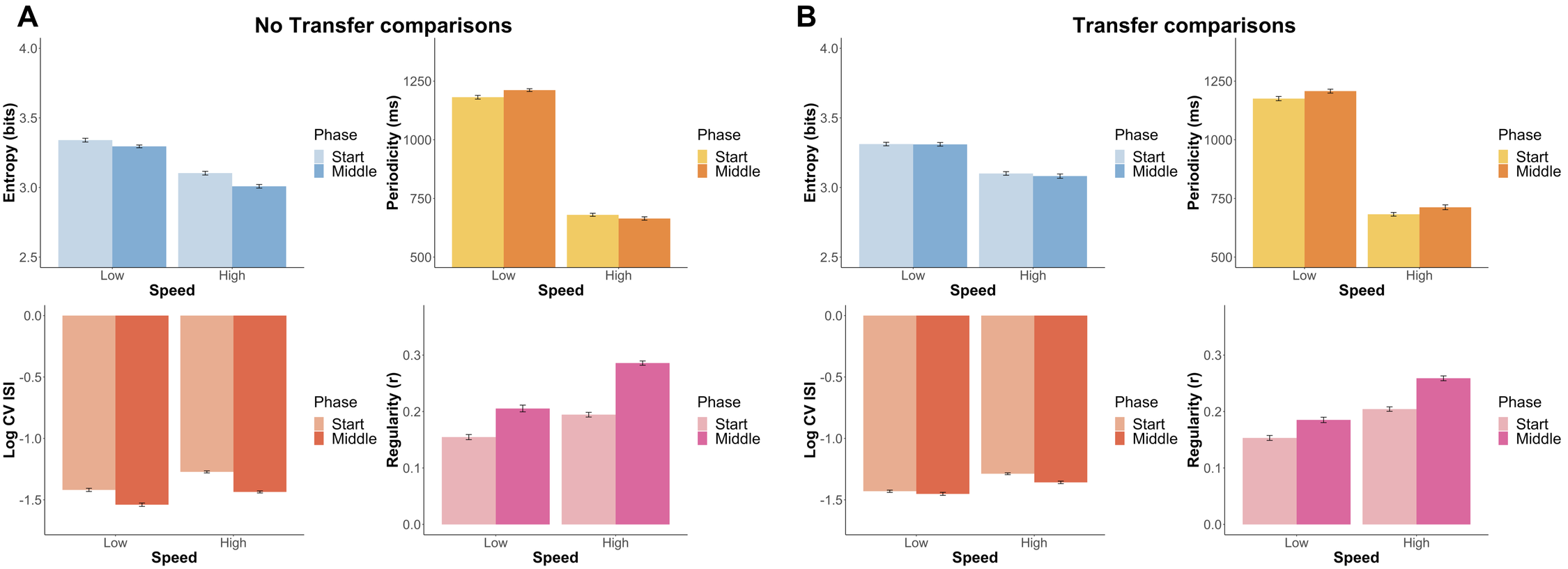

Supplement: S3 Fig — (A) No Transfer phase comparison of ACT-R entropy (blue), shot periodicity (yellow), shot timing variability (red), and shot regularity (pink) in the LLL (low speed only) and HHH (high speed only) conditions. (B) Transfer phase comparison of ACT-R entropy (blue), shot periodicity (yellow), shot timing variability (red), and shot regularity (pink) in the LHL (Low-Start & High-Middle) and HLH (High-Start & Low-Middle) conditions. The bar graph indicates means and standard errors of the mean within speeds and phases. Note that this figure only includes data from the ACT-R model with two trackers and one temperature reset. (TIF) [file pone.0258242.s003.tif]

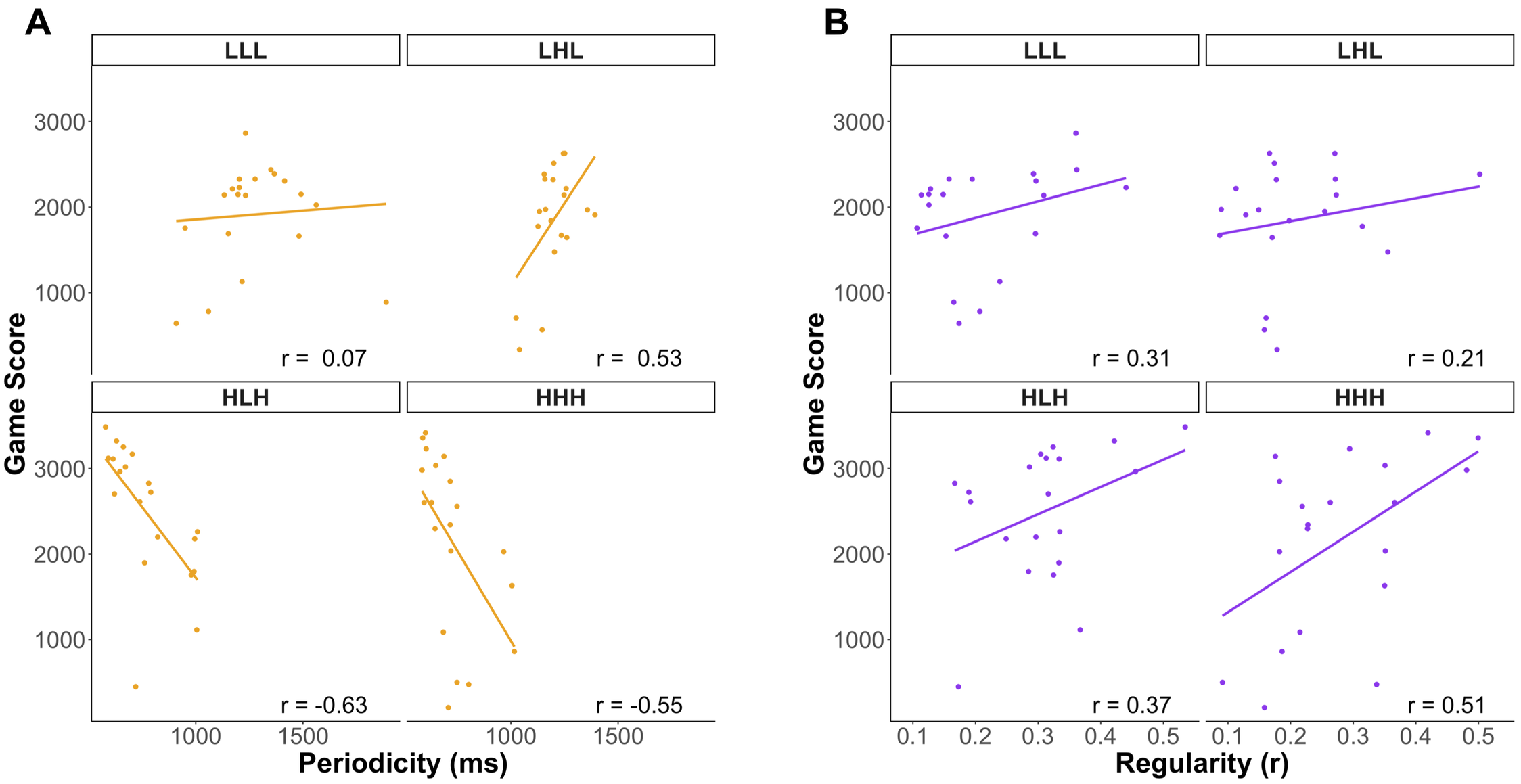

Supplement: S4 Fig — (A) Correlation between human average game score and average shot periodicity. (B) Correlation between human average game score and average shot regularity. (TIF) [file pone.0258242.s004.tif]
