# Supplementary material for: Cognitive & motor skill transfer across speeds: A video game study
Source: PLoS One. 2021 Oct 12;16(10):e0258242. doi: 10.1371/journal.pone.0258242 (PMC8509974; doi:10.1371/journal.pone.0258242)
Supplement: S1 Table — (PDF) [file pone.0258242.s005.pdf]

S1 Table. Table with key ACT-R parameters sorted across function.

	Parameter	*Value*	*Description*
**Retrieval of operators**	:ol	TRUE	optimized learning parameter
	:lf	0.05	latency factor (affects retrieval time)
	:ans	0.01	instantaneous activation noise
	:rt	-1	retrieval threshold
	:bll	nil	base level learning
**Temporal module**	:time-noise	0.005	temporal module's noise
	:time-mult	1.1	multiplier for increasing the pulse length
	:time-master-start-increment	0.011	length of the initial pulse (in s.)
**Motor productions**	:randomize-time	3	allows timing of certain actions to vary
	:dual-execution-stages	T	separate execution stages for the hands
	:motor-feature-prep-time	0.02	time to prepare a movement feature
	:motor-initiation-time	0.02	time to initiate a motor movement
**Utility learning & production compilation**	:ul	TRUE	utility learning
	:epl	TRUE	production compilation parameter
	:egs	0.05	expected gain – utility learning noise
	:alpha	0.2	learning rate in difference learning equation
	:tt	2	threshold time
	:iu	9	initial utilities
**Controller module**	:tracker-decay-method	Exponential	type of decay for game parameters
	:tracker-decay	0.995	decay parameter
	:temp-scale	180	temperature scaling factor
	:initial-temp	1	initial temperature
