# Supplementary material for: Cognitive & motor skill transfer across speeds: A video game study
Source: PLoS One. 2021 Oct 12;16(10):e0258242. doi: 10.1371/journal.pone.0258242 (PMC8509974; doi:10.1371/journal.pone.0258242)
Supplement: S2 Table — (PDF) [file pone.0258242.s006.pdf]

S2 Table. ACT-R model fits across experimental measures in each of the four conditions.

S2A Table. Root mean squared error (RMSE) between humans and ACT-R models with one tracker and one temperature.

**1 Tracker**	**LLL**	**LHL**	**HLH**	**HHH**
Game Score	158.10	701.53	367.06	514.30
Reset count	3.12	8.42	21.41	1.39
Deflation count	1.23	16.16	4.87	4.31
Miss count	5.66	8.43	7.44	4.44
Entropy	0.22	0.25	0.23	0.12
Periodicity	78.12	138.71	133.49	50.24
Log CV ISI	0.46	0.27	0.37	0.48
Regularity	0.04	0.03	0.08	0.04

S2B Table. RMSE between humans and ACT-R models with two trackers and one temperature.

**2 Trackers**	**LLL**	**LHL**	**HLH**	**HHH**
Game Score	117.83	217.53	227.96	506.33
Reset count	3.14	6.22	6.11	1.88
Deflation count	1.22	3.24	4.11	3.92
Miss count	5.47	7.88	7.15	3.98
Entropy	0.21	0.13	0.07	0.13
Periodicity	80.65	58.04	67.78	55.19
Log CV ISI	0.45	0.22	0.26	0.50
Regularity	0.03	0.03	0.03	0.04
