# Supplementary material for: Cognitive & motor skill transfer across speeds: A video game study
Source: PLoS One. 2021 Oct 12;16(10):e0258242. doi: 10.1371/journal.pone.0258242 (PMC8509974; doi:10.1371/journal.pone.0258242)
Supplement: S1 Text — (PDF) [file pone.0258242.s007.pdf]

S1 Text. Instructions provided to participants prior to the *Auto Orbit* experiment.

In this study you will be playing a video game where you control a ship in outer space.

The ship follows a circular orbit around the yellow circle. Press **A** to rotate the ship counter-clockwise and **D** to rotate clockwise.

Launch a missile by pressing **L**. Each missile that hits the yellow circle grows the orange ball inside. When the orange ball completely fills the circle, you can score points by bursting it with a "double shot"—firing two missiles quickly.

If you fire a double shot before the ball completely inflates, it will pop but you will not score points. If you wait too long to hit the circle with a missile, the ball will begin to deflate.

You lose points for each missile that does not hit the yellow circle.

As an extra challenge, the ship may suddenly and unexpectedly rotate. Always be ready.

Note that in some games the ship's orbital speed will be faster or slower and the rate of fire needed to inflate the ball will also be faster or slower.
